# Gesture Recognition with Residual LSTM Attention Using Millimeter-Wave Radar †

**DOI:** 10.3390/s25020469

**Published:** 2025-01-15

**Authors:** Weiqing Bai, Siyu Chen, Jialiang Ma, Ying Wang, Chong Han

**Affiliations:** 1 College of Computer, Nanjing University of Posts and Telecommunications, Nanjing 210023, China; b22040010@njupt.edu.cn (W.B.); 1022040914@njupt.edu.cn (S.C.); b22040007@njupt.edu.cn (J.M.); b22040008@njupt.edu.cn (Y.W.); 2 Jiangsu High Technology Research Key Laboratory for Wireless Sensor Networks, Nanjing University of Posts and Telecommunications, Nanjing 210023, China

**Keywords:** millimeter-wave radar, gesture recognition, signal preprocessing, deep learning

## Abstract

Gesture recognition technology based on millimeter-wave radar can recognize and classify user gestures in non-contact scenarios. To address the complexity of data processing with multi-feature inputs in neural networks and the poor recognition performance with single-feature inputs, this paper proposes a gesture recognition algorithm based on **R**esNet **L**ong Short-Term Memory with an **A**ttention Mechanism (RLA). In the aspect of signal processing in RLA, a range–Doppler map is obtained through the extraction of the range and velocity features in the original mmWave radar signal. Regarding the network architecture in RLA, the relevant features of the residual network with channel and spatial attention modules are combined to prevent some useful information from being neglected. We introduce a residual attention mechanism to enhance the network’s focus on gesture features and avoid the impact of irrelevant features on recognition accuracy. Additionally, we use a long short-term memory network to process temporal features, ensuring high recognition accuracy even with single-feature inputs. A series of experimental results show that the algorithm proposed in this paper has higher recognition performance.

## 1. Introduction

Gesture recognition is one of the most commonly used and effective methods of human–computer interaction [1]. Current gesture recognition technologies are primarily categorized into three types: visual image-based [2], inertial sensor-based [3], and bio-signal-based [4] technologies. While these methods achieve high accuracy, they also present challenges such as user privacy concerns, operational complexity, and constraints on user behavior. To address these issues, researchers have increasingly focused on gesture recognition using millimeter-wave radar technology. Radar-based systems offer advantages such as resilience to lighting conditions, a strong penetration capability, low power consumption, and a compact design, making them a prominent research area in gesture recognition [5,6].

However, millimeter-wave radar-based gesture recognition faces two primary challenges. First, processing radar signals is complex, as gesture signal extraction can be impeded by factors like DC signal interference, multipath effects, and environmental noise. Second, constructing deep learning networks for this purpose requires handling complex, multi-dimensional feature data; relying on single-feature inputs can lead to suboptimal recognition performance, ultimately impacting gesture recognition accuracy [7].

In response to these challenges, this paper proposes a gesture recognition algorithm based on ResNet–Long Short-Term Memory with an attention mechanism. RLA leverages the advantages of Frequency Modulated Continuous Wave (FMCW) millimeter-wave radar, such as its compact antenna size, low power usage, and high range resolution, integrating these benefits with deep learning techniques for improved gesture recognition.

The main contributions of our work are summarized as follows.

- In the signal preprocessing stage, this paper adopts a simple and straightforward background subtraction method to filter out noise, including static clutter, multipath reflections, and DC components after Fourier transformation. This approach is intentionally uncomplicated, ensuring the preservation of gesture signals without adding processing complexity. Furthermore, range–Doppler maps (RDM) are created based on gesture velocity and range data, serving as clear and efficient inputs to the network model.
- In terms of deep learning architecture, this paper combines the strengths of multiple network types to maximize performance. To fully utilize network capabilities while avoiding issues like gradient vanishing, a Residual Long Short-Term Memory (ResNet-LSTM) network is employed for processing input features. Additionally, a residual attention module is integrated into the network that directs attention to critical variations in gesture features, significantly boosting gesture recognition accuracy. This module sequentially generates attention maps across both channel and spatial dimensions, then multiplies these maps with the network’s input feature maps to create new feature maps. These enhanced maps are further processed for feature extraction, resulting in a notable performance gain.

The remainder of this paper is organized as follows. Section 2 reviews related work in the field of gesture recognition, particularly focusing on FMCW radar-based approaches. Section 3 provides an overview of the proposed RLA algorithm, detailing the signal acquisition, preprocessing steps, and the deep learning architecture, including the ResNet-LSTM network and the residual attention module. Section 4 presents the experimental setup and results, demonstrating the effectiveness of the proposed method. Finally, Section 5 concludes the paper and discusses potential future work.

## 2. Related Work

For the current study, gesture recognition systems can be classified into four main categories according to the media used: visual image-based, inertial sensor-based, bio-signal-based, and radar-signal-based gesture recognition. Among radar signals, FMCW radar is gradually gaining attention from researchers given its low power consumption, high system integration, high resolution, and strong ability to extract micro-motion information from targets.

Currently, gesture recognition based on FMCW radar has made significant progress. One of the most representative cases is the Soli radar developed by Google ATAP’s Touchstone team [8]. They embarked on a comprehensive research and development effort, starting with the construction of sensor architecture, and independently developed a millimeter-wave radar chip. They evaluated the chip’s performance by establishing relevant datasets.

Smith et al. [9] developed a gesture recognition technique using a 60 GHz mmWave radar sensor for in-car infotainment control, aiming to provide a safe and intuitive interface to reduce driver distraction. They extracted specific gesture features and built a machine learning engine capable of real-time recognition, considering user requirements and in-car environmental constraints. The article presents accuracy results and offers recommendations for further research and improvements in this domain. Yan et al. [10] explored various radar signal inputs for gesture recognition, including RDI, RAI, DAI, and Micro-Doppler spectrograms. They proposed a semi-supervised learning framework that combines a small set of labeled data from the source domain with a large amount of unlabeled data from the target domain. By applying the π-model and data augmentation techniques on mmWave signals, they achieved domain-independent gesture recognition. Extensive experiments on a public mmWave gesture dataset demonstrated the effectiveness of their system, advancing contact-free and privacy-preserving gesture recognition. Ehsanfar et al. [11] analyzed the ROC of FMCW, OFDM, and hybrid radars in the IEEE 802.11bd frame. They compared detection performance for FMCW, OFDM, and hybrid systems, showing a 5–7 dB gain for the hybrid radar and demonstrating its feasibility for joint communication and radar. Reddy et al. [12] utilized mmWave signals in cooperative localization to achieve sub-meter accuracy in linear topology applications. Their work derived performance limits and closed-form expressions for position error bounds, highlighting that the number of anchor nodes has a greater impact on localization accuracy than the number of antenna elements. Antes et al. [13] explored the relationship between radar parameter settings and gesture velocity. The study involved 25 participants performing 15 predefined gestures and 1 custom movement to determine the key velocity parameters necessary for gesture recognition. It concluded that the gesture set and application setup are crucial design choices, with expected velocities derivable from a few test participants. This work provides valuable insights into radar parameterization for gesture recognition applications. Molchanov et al. [14] utilized a 2D Fast Fourier Transform (2D-FFT) on collected radiofrequency signals to obtain range–Doppler maps (RDMs) that contained information about gesture range and velocity, which were employed to represent gestures. Mao et al. [15] proposed a low-complexity hand gesture recognition framework using a distributed multiple FMCW radar system, leveraging synthesized motion velocity vectors and an LSTM network to achieve 98% accuracy on a 1600-sample dataset. These contributions highlight significant advancements in FMCW radar-based gesture recognition research. Si-Jung et al. [16], on the other hand, acquired RDM images directly from the raw signals of an FMCW radar. They generated multiple features based on the obtained images in conjunction with a feature selection algorithm inspired by quantum evolution, achieving gesture recognition. Gan et al. [17] proposed a method for processing the acquired range–Doppler images, introducing a range–Doppler matrix focus (RDMF) for dimensionality reduction. Wang et al. [18] introduced two types of feature maps, the range–time map (RTM) and Doppler–time map (DTM), as inputs to a network, thus constructing a gesture recognition system. These advancements collectively demonstrate the evolving landscape of FMCW radar-based gesture recognition research. Cardillo et al. [19] proposed an advanced radar-based solution for monitoring sedentary behavior and recognizing physical activities in the workplace. By leveraging microwave radar technology, they demonstrated the ability to track breathing activity, position, and human micro-Doppler signatures, enabling the detection of activities such as standing, sitting, and exercising while preserving privacy and ensuring cost-effectiveness. Zhang et al. [20] analyzed and processed gesture echoes received using a 9.8 GHz radar device. They obtained micro-Doppler feature maps corresponding to different gestures and utilized support vector machines (SVMs) for classification. They achieved relatively high accuracy for four predefined gestures. In order to solve the problem of micro-motion gesture features being not obvious and difficult to identify, Bao et al. [21] proposed a gesture recognition method based on multi-scale fusion deep network, which is mainly composed of 2D convolution modules, i.e., a multi-scale fusion module and attention mechanism module. The multi-scale fusion module consists of three residual blocks of different scales, which can obtain different sizes of receptive fields and multi-scale features, as a way to identify many different gestures, and the experimental results show that the method is simple to implement and does not require complex data preprocessing.

Research on millimeter-wave radar-based gesture recognition has made significant progress, particularly in data preprocessing, feature extraction, and recognition. However, challenges persist, especially in managing environmental noise, as current algorithms often face issues with complexity and limited effectiveness. Moreover, recognition accuracy remains inconsistent when relying on single-feature inputs. To overcome these limitations, this paper presents a gesture recognition algorithm that utilizes a residual attention mechanism to improve performance.

## 3. The Proposed RLA Algorithm

Gesture feature information is captured by transmitting FMCW signals and receiving their echoes through a millimeter-wave radar platform. The signal generator produces linear frequency-modulated (FM) pulses, with frequency increasing linearly over time, which are transmitted via the radar’s antenna. These pulses reflect off the gesture, and the radar’s receiving end captures the echo signals. After mixing, an intermediate frequency (IF) signal is produced, which is then processed to extract the gesture’s feature information.

**Figure 1:**
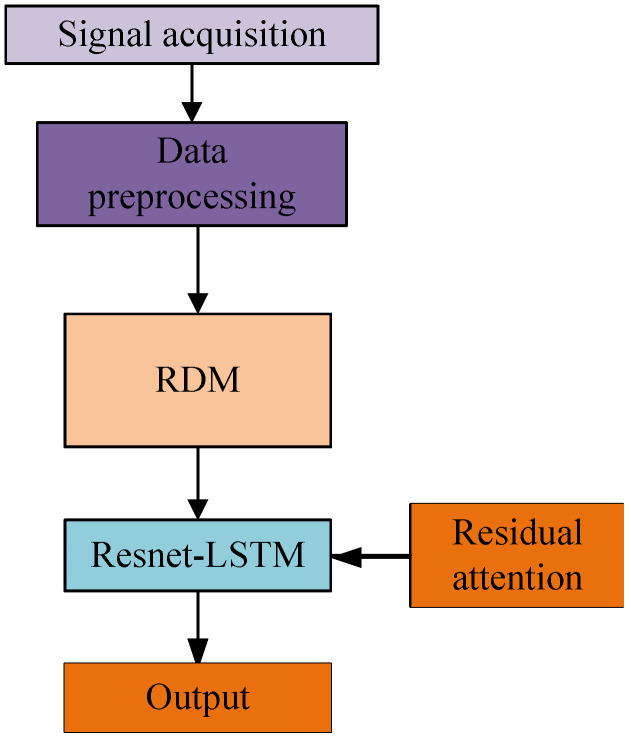
Overall structure of gesture recognition algorithm.

The architecture of the proposed RLA gesture recognition is shown in Figure 1. After the acquisition, the signal is preprocessed by the dataset to obtain the range and velocity measurements of the gesture, and the range–Doppler image containing the gesture features is generated by combining the two different information, which is predicted by the ResNet-LSTM.

### 3.1. Gesture Feature Extraction and Clutter Removal

This section outlines the process of acquiring IF signals and gesture velocity and range information in radar analysis. Due to the presence of direct current and noise in IF signals, which can cause confusion in gesture echoes, a background subtraction method is proposed to mitigate their impact.

#### 3.1.1. Acquisition of IF Signals

The transmit signal can be denoted as follows:

(1) $$S_{T}=A_{T}\cos\left[2\pi f_{c}t+2\pi \int_{0}^{t}f_{T}\left(\tau \right)\;d\tau \right]$$

where $A_{T}$ denotes the transmit signal amplitude, $f_{c}$ is the center frequency, and $f_{T}\left(\tau \right)$ denotes the frequency of the transmit signal. The received echo signal of the hand gesture can be denoted as follows:

(2) $$S_{R}\left(t\right)=A_{R}\cos\left[2\pi f_{c}\left(t-\tau \right)+2\pi \int_{0}^{t}f_{R}\left(\tau \right)\;d\tau \right]$$

where $A_{R}$ represents the amplitude of the echo signal. The mixing signal can be obtained from the transmit and echo signals:

(3) $$S_{M}\left(t\right)=S_{T}\left(t\right)\cdot S_{R}\left(t\right)$$

Finally, the mixed signal is passed through a low-pass filter to obtain the IF signal containing the gesture feature information:

(4) $$S_{\mathrm{IF}}\left(t\right)=\frac{1}{2}A_{T}A_{R}\cos\left[2\pi (f_{c}\tau +\frac{B}{T}\tau t-f_{d}t)\right]$$

where $S_{\mathrm{IF}}\left(t\right)$ is the IF signal, τ is the time delay, B denotes the signal bandwidth, and T represents the modulation period. The distance and speed of the gesture target can be calculated based on the time delay of the transmitted and received signals contained in the IF signal.

#### 3.1.2. Measurement of Gesture Range and Velocity

The range between the radar and gesture can be calculated using the time delay:

(5) $$R=\frac{c\tau }{2}$$

where *R* is the range and *c* is the speed of light. Since the time delay cannot be obtained directly, it is necessary to estimate the range by the frequency of the IF signal. The relationship between frequency and time of the IF signal is as follows:

(6) $$\frac{f_{\mathrm{IF}}}{\tau }=\frac{B}{T}$$

Thus, the time delay τ can be expressed as follows:

(7) $$\tau =\frac{f_{\mathrm{IF}}T}{B}$$

After performing the FFT on the transmitted signal and the received signal, objects at the same distance exhibit identical peaks in frequency. However, due to differences in dephasing, targets with different velocities can be distinguished by the phase difference ω. The expression for ω is given by the following:

(8) $$\omega =2\pi f_{IF}\Delta \tau$$

The velocity for the target can be deduced as follows:

(9) $$v=\frac{\lambda \omega }{4\pi T_{c}}$$

**Figure 2:**
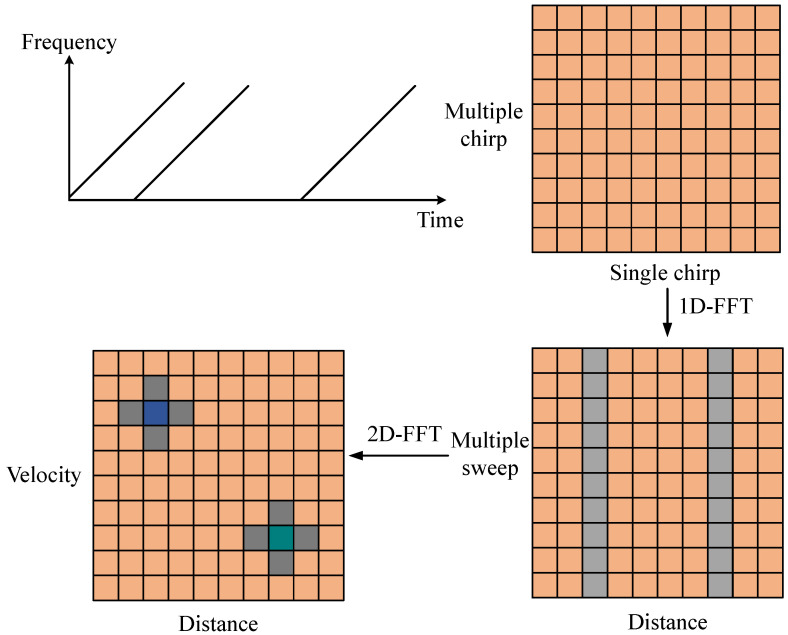
2D-FFT Processing Flow.

After processing the radar signal with a 2D-FFT, a range–Doppler feature map can be constructed, as illustrated in Figure 2. The ADC sampling backend data are stored in a two-dimensional matrix, organized by chirps. Multiple chirp times are referred to as the slow-time domain, while a single chirp time is called the fast-time domain.

The intermediate frequency (IF) signal spectrum can be obtained by performing a Fourier transform on the radar chirp signal within a single chirp time (the fast-time domain) in Figure 2. This is achieved by conducting range-FFT along each row.

By combining Equations (5) and (7), one can identify the frequency points of the targets by searching the spectra of different separated peaks, thereby obtaining the distance information between the targets and the radar.

For processing in the slow-time domain, the spectra of multiple fast-time domains need to be accumulated first. Subsequently, a Fourier transform is performed on the slow-time dimension of the accumulated result—that is, velocity-FFT is conducted along each column. After coupling, this process yields the range–Doppler map (RDM). In this paper, the radar sampling frequency ($f_{c}$) is 3 MHz, and the maximum detectable range ($d_{max}$) of the radar is 6.4 m.

#### 3.1.3. Clutter Removal and Heatmap Optimization

Since static object reflections remain relatively fixed in the range–Doppler map, we use background subtraction to eliminate static clutter. During the dataset recording process, signals of the empty background without gestures are captured, and then the range–Doppler images of the empty background alone are plotted. Static clutter is then removed using background subtraction, as illustrated in the following example:

(10) $$du_{pq}=du_{pq}-\mathrm{mean}\left(p\right)$$

where $du_{pq}$ is the data in row *p*, column *q*, and the mean (*p*) is the mean value of row *p* in the empty background. In addition to clutter interference, spectral leakage due to the fast Fourier transform (FFT) of the radar signal results in poor spectral resolution. To address this issue, we apply a Hanning window before the FFT operation.

(11) $$S_{d}\left(N,P,f\right)=S\left(N,P,f\right)\cdot \mathrm{Hanning}\left(N\right)$$

For the velocity, it is as follows:

(12) $$S_{V}\left(N,P,f\right)=S_{d}\left(N,P,f\right)\cdot \mathrm{Hanning}\left(P\right)$$

The processed range–Doppler maps can better highlight the velocity and range information during the gesture movement, and the consecutive radar frames can reflect the movement trajectory of the gesture. Optimising the radar signals during the signal processing stage can improve the recognition accuracy of the gesture recognition system to a certain extent, and also this paper is an important part of the gesture recognition process.

### 3.2. Network Model Based on Residual Attention Mechanism

#### 3.2.1. Construction of Residual Networks

To address the issue of network degradation caused by increasing network depth, He et al. [22] proposed ResNet, which introduced residual modules. These modules enable the network to bypass several layers by utilizing shortcut connections, allowing the output of earlier layers to be directly propagated to later layers. This architecture effectively mitigates the vanishing gradient problem during backpropagation, facilitating the training of very deep networks. ResNet has achieved significant breakthroughs, particularly in image classification tasks, where it has become a cornerstone model. Numerous classification-related applications based on ResNet have been developed in recent years, showcasing its robustness and versatility [23].

Gesture recognition should be considered a classification problem. To address this, we adopt the ResNet50 architecture as the neural network’s backbone. Experimental results demonstrate that this architecture strikes the optimal balance between accuracy and model complexity, making it the most suitable foundational framework.

#### 3.2.2. Introduction of Attention Mechanisms

Attention mechanisms were initially proposed in the field of computer vision, with the core idea of enabling networks to focus on the most relevant regions, thereby enhancing their learning capabilities. Over time, attention mechanisms have been widely applied across various domains, showcasing remarkable performance [24]. Vaswani et al. [25] provided an abstract definition:

(13) $$\mathrm{Attention}\left(Q,K,V\right)=\mathrm{softmax}\left(\frac{QK^{T}}{\sqrt{d_{K}}}\right)\cdot V$$

where *Q* stands for query, *K* stands for key, and *V* stands for value, all three are vectors, and the attention mechanism is looking to establish a mapping relationship from *Q* to the key–value pair K−V. The general attention mechanism can be divided into channel and spatial attention mechanisms.

**Figure 3:**
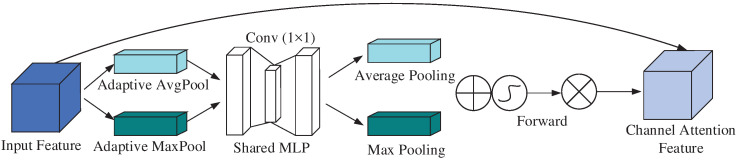
Structure of the channel attention.

The channel attention mechanism compresses the channel information of the input feature map. Its main function is to adaptively adjust the weights of different channels in the feature map, enhancing the network’s focus on key channel information while suppressing the influence of less important channels. Without significantly increasing computational cost, this mechanism optimizes feature representation, thereby improving the accuracy of classification tasks. Specifically, it first performs average pooling and max pooling operations on the feature map channels, then passes the pooled results through a shared network to adjust the channel weights and obtain fused features. These fused features are then processed by an activation function to generate weight parameters, which are finally multiplied by the input feature map to obtain new features. The structural principle is shown in Figure 3.

**Figure 4:**
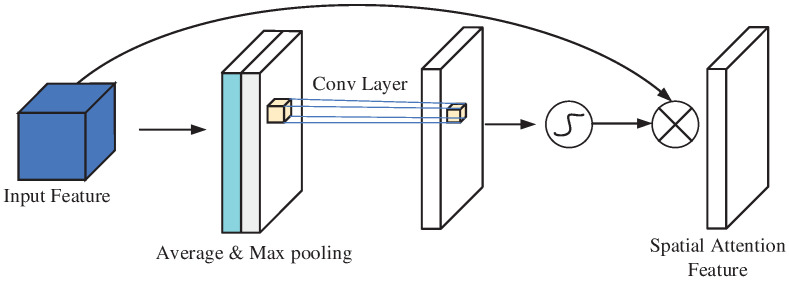
Structure of spatial attention.

The spatial attention mechanism enhances the model’s focus on the most informative regions of the feature map, effectively improving feature representation. By leveraging average pooling and max pooling along the channel axis, it generates two weight vectors, which are combined to form a descriptor. This descriptor is then convolved and processed using a normalization function to derive the weight parameters. These parameters are subsequently multiplied with the input feature map to emphasize critical spatial regions and suppress irrelevant ones, resulting in refined features. The structural schematic is illustrated in Figure 4.

**Figure 5:**
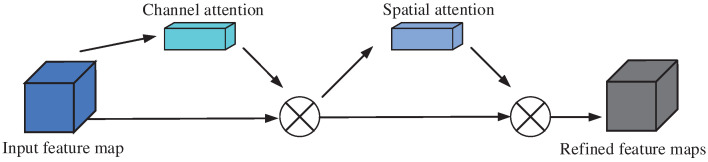
Schematic diagram of CBAM structure.

To better leverage the strengths of attention mechanisms, we established the Convolutional Block Attention Module (CBAM) architecture, which combines the advantages of both channel and spatial attention mechanisms. CBAM first applies channel attention to adaptively highlight the most informative feature channels. It then employs spatial attention to refine the focus on critical spatial regions of the feature map by generating descriptors. By sequentially integrating these two attention mechanisms, CBAM effectively enhances feature representation, improving model performance with minimal computational overhead. The structural schematic is shown in Figure 5.

**Figure 6:**
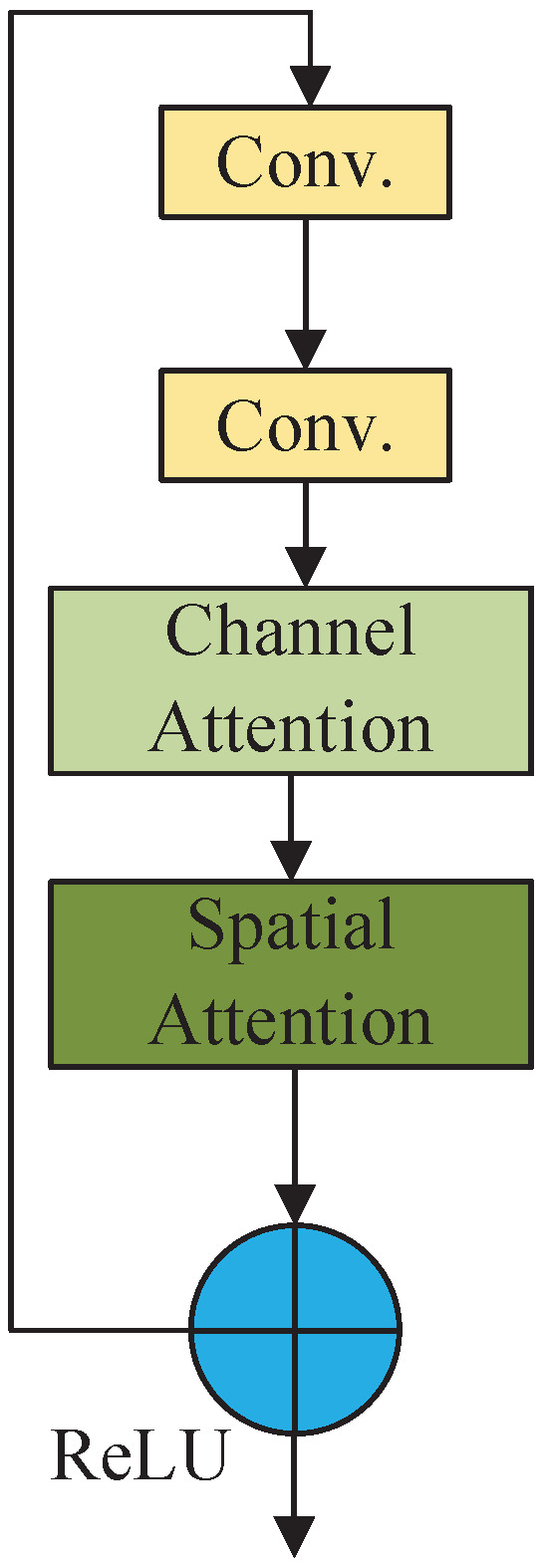
Structure of residual attention module.

The module is placed within the residual attention module composed of residual blocks in the residual network to make the network pay more attention to the gesture feature information in the input feature map. The implementation of this module involves adding a convolutional block attention module after the convolutional layer of the first residual block in the residual network. The attention mechanism is achieved through the interaction between the residual block and the attention module, as illustrated in Figure 6. The input feature map goes through two layers of convolutional layers to extract features. Subsequently, it enters the channel attention and spatial attention module. The results obtained from these modules are added to the input feature map and then activated by the ReLU function for output.

#### 3.2.3. Network Architecture

**Figure 7:**
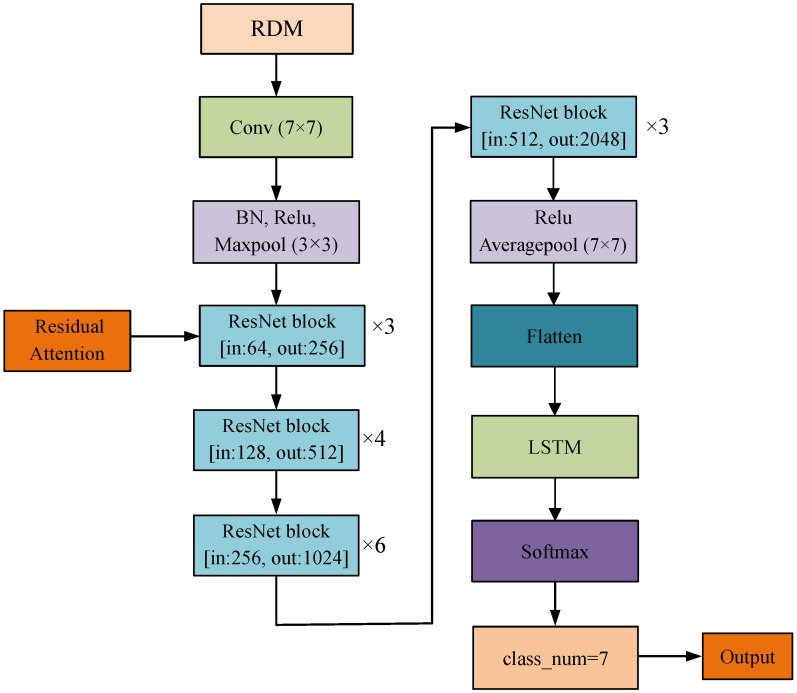
Network architecture based on residual attention mechanism.

In summary, we propose a network model based on the residual attention mechanism, as depicted in Figure 7. The proposed network architecture takes the range–Doppler map (RDM) as input and incorporates a residual attention mechanism to enhance feature extraction. This mechanism processes features through two convolutional layers, followed by channel attention and spatial attention modules, which selectively emphasize important gesture-related features while suppressing irrelevant ones. The combined outputs are added to the original feature map and activated using a ReLU function, enabling the network to focus more effectively on gesture-specific information.

The residual attention mechanism is seamlessly embedded within the residual blocks of ResNet50, ensuring efficient integration without compromising the network’s performance. By dynamically adjusting feature weights, the attention mechanism allows the network to adapt to varying conditions and prioritize critical information, enhancing the overall representation of spatial features.

The output from the attention-enhanced ResNet50 is passed through fully connected layers to an LSTM network, which captures temporal dependencies in the sequential data. This combination of the residual attention mechanism for spatial features and the LSTM network for temporal modeling provides a comprehensive understanding of gestures. By leveraging both spatial and temporal information, the architecture achieves superior accuracy in gesture recognition, demonstrating the effectiveness of its design.

## 4. Experiment and Evaluation

### 4.1. Experimental Setup and Parameters Setting

The hardware system for mmWave radar signal acquisition and validation used in this study consists of two modules: the millimeter-wave radar evaluation board (IWR1843BOOST) and the DCA1000 evaluation board (DCA1000EVM) (Texas Instruments, Dallas, TX, USA).

The radar-related parameter settings for the gesture recognition system are summarized in Table 1.

In our experiments, the LSTM model was configured with a hidden layer dimension of 1024, a learning rate of 0.001, a dropout rate of 0.1, and a batch size of 32.

### 4.2. Gesture Dataset

In this section, we evaluate and compare the network model based on the Resnet-LSTM attention mechanism using two gesture datasets: a self-created gesture dataset and the Google Soli dataset [26], both collected with mm-wave radar. This evaluation aims to validate the model’s recognition accuracy and generalization capabilities.

#### 4.2.1. Self-Constructed Datasets

**Figure 8:**
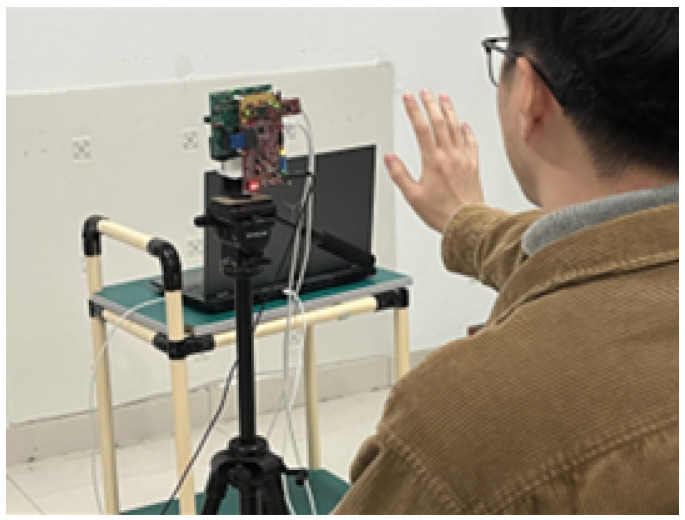
Gesture data acquisition.

The primary application scenario of this study focuses on enabling drivers to interact with in-car smart devices using hand gestures, thereby reducing the risk of distracted driving accidents. Considering the driver’s posture and the in-car environment during vehicle operation, the dataset was collected under conditions that closely simulated real driving scenarios. Specifically, experimental subjects were seated in front of the radar, as illustrated in Figure 8, with hand gestures recorded at a distance of approximately 30 cm from the radar. This setup was designed to replicate the driver’s actual interaction environment during data recording, ensuring the relevance and practicality of the dataset for the intended application.

**Figure 9:**
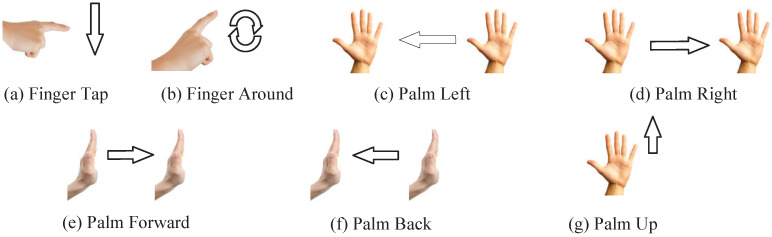
Types of gestures.

For the driving process considered in this paper, we designed a total of seven kinds of gestures, respectively: finger click, finger around the circle, palm left, palm right, palm forward push, palm back pull, and palm up row. Each specific gesture action is shown in Figure 9.

Its application scenarios in the driving control are as follows:

1. Finger Tap, a click in the air with the index finger to determine the opening of the software on the selected multimedia device.
2. Finger Around, the index finger makes a circling motion in space to adjust the volume level of a multimedia device.
3. Palm Left, five fingers together and move them from right to left to swipe the interface left or select software on a multimedia device.
4. Palm Right, five fingers together and move from left to right to swipe right on a multimedia device or select software.
5. Palm Forward, five fingers together and pushing the palm in a direction away from the driver’s body, used to wake up the multimedia device.
6. Palm Back, five fingers together and push the palm in the direction close to the driver’s body, used to lock the multimedia device to prevent accidental touch from occurring.
7. Palm Up, five fingers together to slide the palm upwards to exit the software currently open on your multimedia device and return to the software selection screen.

The 7 gestures can effectively fulfill the driver’s need to interact with in-car multimedia devices while driving. In total, this study recruited 10 users, including 7 males and 3 females, for the recording of these 7 gestures, with each gesture repeated 20 times. Additionally, the dataset includes data from two additional users performing the same 7 gestures. The gesture data from these two users will not be used in model training but will serve as a test set to evaluate the model’s ability to recognize gestures from new users.

#### 4.2.2. Soli Gesture Dataset

The Soli dataset [26] is a gesture dataset based on millimeter-wave radar, developed by Google’s Soli team to support research on the Soli chip and evaluate the performance of end-to-end gesture recognition network models. The dataset is characterized by two key features:

1. Gesture Design: The gestures primarily involve fine movements of the fingers and wrist joints, driven by small muscle groups. This design minimizes user fatigue during prolonged gesture interactions, making the dataset suitable for practical applications.
2. Dynamic Gesture: Most gestures in the dataset are dynamic in order to align with the signal characteristics of millimeter-wave radar. The dataset includes 11 distinct gestures, recorded by 10 participants. The original range–Doppler maps were captured at a frequency of 40 Hz. Preprocessing steps include normalization and background removal using a per-pixel Gaussian model. Each participant performed each gesture 25 times, resulting in a total of 11 × 25 × 10 = 2750 gesture sequences.

#### 4.2.3. Experimental Results and Comparison

Firstly, we discuss comparing the performance of ResNet50 with other residual networks on the self-built dataset. We experimentally compare the four ResNet networks, ResNet18, ResNet34, ResNet50, and ResNet101, and train the networks using the self-built dataset, and also test them on the trained models, with the training set and test set configuration of 8:2; the test results were obtained as shown in Table 2.

From the comparative results, it is evident that the recognition accuracy improves progressively with the increasing depth of networks, as observed in ResNet18, ResNet34, and ResNet50. This trend suggests that as the depth of the network increases, the recognition accuracy also tends to improve, up to a certain point. However, with ResNet101, the recognition accuracy decreases compared to ResNet50. This indicates that beyond a certain depth, further increasing the network can lead to degradation. And excessive depth may reduce recognition performance. To optimize classification accuracy and fully exploit the benefits of residual networks at considerable depth, this study chooses ResNet50 as the foundational network and incorporates an attention mechanism to develop the ResNet-LSTM model.

(1) Performance evaluation on self-constructed gesture dataset

**Figure 10:**
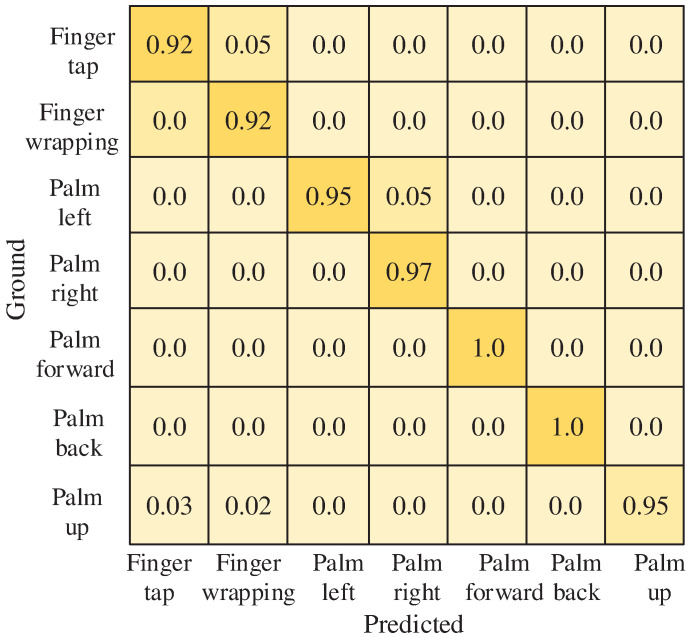
Confusion matrix for the seven gestures.

The recorded gesture dataset, comprising data from 10 users, is divided into training and test sets with a ratio of 8:2. The RLA gesture recognition algorithm’s network model, as proposed in this paper, is first trained using this dataset. Figure 10 presents the confusion matrix illustrating the algorithm’s recognition accuracy for different gestures within the dataset. The depth of the color represents the level of accuracy. From this confusion matrix, it is evident that the trained model effectively distinguishes between gestures, achieving accurate recognition for movements like pushing the hand forward and pulling the hand backward. Additionally, the model demonstrates strong recognition performance for other movements within the self-constructed dataset.

Then, based on this dataset the present algorithm was compared with other gesture recognition algorithms and the comparison results for each gesture action are shown in Table 3.

Table 3 shows that the RLA gesture recognition algorithm achieves 96.1% recognition accuracy for the self-built dataset. Compared to other algorithms, RD-T uses both RTM and DTM feature maps as inputs to the model. While it extracts features related to gesture velocity and range, it does not fully leverage the relationship between velocity and range information. These two features are treated independently, resulting in a lower gesture recognition rate. RDMF introduces a focused feature map for RDM, acting as an attention mechanism. It feeds the RDMF cubic module, containing both range and velocity information, into a 3D-CNN for training. This method is used to extract useful information, but as it only takes some RDM maps as input, there may be a loss of certain feature information, leading to an overall lower recognition rate.

MF-FTN extracts features from RDM and ATM separately using two networks, and then employs a feature fusion method for gesture recognition and classification. This approach does not particularly focus on regions of the map containing more feature information and is also influenced by network depth, resulting in a lower network recognition rate.

In addition to the accuracy of the model, the recall rate, precision rate, and F1 value are also important indicators to react to the performance of a classification model. The recall rate can react to the proportion of the number of gestures retrieved by the model to the number of all relevant gestures in the test set can be used to verify the model’s checking effect, and the precision rate can be used to verify the model’s checking effect.

In order to better characterise the performance metrics of the model in terms of recall and precision, the F1 value of the model is also computed, whose value can be obtained by averaging the precision and recall in a reconciled manner. The three metrics for different network structures during the ablation experiments are shown in Table 4, from which it can be seen that the RLA algorithm has some advantages in all three metrics.

Finally, ablation experiments were also conducted on the self-built dataset for the present algorithm; three network structures, ResNet50, ResNet50+LSTM, and the RLA network model proposed in this paper, were used for comparison and the recognition accuracy values for different gestures in the dataset are shown in Table 5.

It is observed that employing a single ResNet50 network for direct gesture recognition yields an average accuracy of only 87.1%. Enhancements to the ResNet50 network lead to improved gesture recognition accuracy, notably through the extraction of temporal information via the LSTM network. This incorporation of LSTM significantly boosts the model’s performance by capturing the sequential dependencies in the input data. Furthermore, integrating a residual attention mechanism markedly enhances the network’s focus on gesture features, leading to a further increase in gesture recognition accuracy. This combination of LSTM for temporal insights and attention mechanisms for feature emphasis results in a substantial performance improvement.

(2) Experimental results on the soil public dataset

To validate the generalization ability of this algorithm, we conducted comparative experiments using the publicly available Soli gesture dataset [26] and the CNN+LSTM end-to-end gesture recognition algorithm proposed by the Soli team, referred to as End to End (ETE). We evaluated the performance of our proposed ResNet-LSTM attention mechanism-based gesture recognition network architecture, RLA, using the Soli dataset. For the experiments with the Soli dataset, both the Google ETE model and our RLA model had an RDM as input. Additionally, to ensure a fair comparison, we set experiment-related parameters, including a 5:5 ratio of training to testing data split and 50 epochs for iteration, to be the same as those used for ETE. This ensures that the comparative experiments were conducted under the same parameters, with the same input, and using the same dataset. The recognition accuracy for the 11 gesture actions in the dataset is shown in Table 6.

We can see that the average recognition accuracy of the RLA network model for the 11 gestures is about 95.2%, which is higher than the average 87% accuracy of the ETE model given in the paper, indicating that the model proposed in this paper has some advantages in recognition accuracy. From the above experiments, it can be seen that the gesture recognition algorithm based on the ResNet-LSTM attention mechanism proposed in this paper has a good performance in terms of generalization ability of the algorithmic model and accuracy of gesture recognition.

## 5. Conclusions

In this paper, we present a gesture recognition method based on the TI millimeter-wave radar platform. Since our approach relies on mmWave radar to obtain the position and velocity information of the target, it does not perform well when dealing with overlapping gestures. This method employs background subtraction to remove static reflections and noise interference from the environment. Furthermore, it integrates the concept of residual networks with dual attention mechanisms, creating a robust residual attention framework that significantly enhances accuracy in gesture recognition with single-feature inputs. The incorporation of LSTM networks plays a crucial role in capturing temporal dependencies, while the dual attention mechanisms focus on extracting and refining critical gesture features. Through theoretical analysis and comparative experiments, the superiority of this algorithm in gesture recognition tasks is robustly demonstrated, highlighting the substantial improvements attributed to the LSTM and attention mechanisms. In the future, we plan to enhance the robustness of the algorithm to various environmental conditions, optimize it for real-time applications, and explore multi-modal fusion with additional sensors to further improve gesture recognition accuracy.

## Figures and Tables

**Table 1:** Radar parameter settings.

Parameters	Value	Parameters	Value
Sweep range	60∼64 GHz	Bandwidth	4 GHz
Range resolution	3.75 cm	Angular resolution	29°
Sampling point	256	Sampling rate	1 Msps
Frame rate	64	Frame rate	25 frames/s
TX (Transmit)	3	RX (Receive)	4

**Table 2:** Test results of different ResNet architectures on self-built dataset.

Model	Training Acc (%)	Test Acc (%)
ResNet18	88.32	87.12
ResNet34	88.47	87.23
ResNet50	89.74	89.11
ResNet101	87.93	88.47

**Table 3:** Different algorithms on self-built dataset.

	Recognition Acc. (%)							
	1	2	3	4	5	6	7	Avg.
RD-T [18]	90	92.5	92.5	95	95	92.5	95	93.2
RDMF [17]	90	92.5	95	92.5	100	97.5	92.5	94.3
MF-FTN [27]	95	97.5	92.5	92.5	95	97.5	95	95
RLA (Ours)	92.5	92.5	95	97.5	100	100	95	96.1

**Table 4:** F1 values for different gesture algorithms.

Model	Recall Rate	Accuracy	F1 Value
RD-T [18]	92.80%	94.40%	93.60%
RDMF [17]	93.80%	94.30%	94.10%
MF-FTN [27]	93.50%	95.30%	94.40%
RLA (Ours)	96.00%	96.10%	96.10%

**Table 5:** Experimental results of ablation experiments.

	Recognition Acc. (%)							
	1	2	3	4	5	6	7	Avg.
Resnet50 only	87.5	85	82.5	85	95.5	92.5	85	87.6
Resnet50 + LSTM	92.5	90	92.5	92.5	97.5	100	92.5	93.9
RLA (Ours)	92.5	92.5	95	97.5	100	100	95	96.1

**Table 6:** Results of RLA on Soli dataset.

Recognition Acc. (%)												
	(a)	(b)	(c)	(d)	(e)	(f)	(g)	(h)	(i)	(j)	(k)	(avg)
ETE [26]	67.7	71.1	77.8	94.5	84.8	98.5	98.6	88.9	94.9	89.6	92.6	87.2
RLA (Ours)	86.8	87.9	93.4	97.6	98.2	96.9	100	100	96.8	94.1	95.6	95.2

## Data Availability

The data presented in this study are available upon request from the corresponding author.
